# Introduction to special issue on Advances in Brain Imaging

**DOI:** 10.1007/s11682-019-00050-6

**Published:** 2019-03-02

**Authors:** Evan D. Morris

**Affiliations:** 1 Department of Radiology and Biomedical Imaging, Yale University, New Haven, CT USA; 2 Department of Biomedical Engineering, Yale University, New Haven, CT USA; 3 Department of Psychiatry, Yale University, New Haven, CT USA

## Symposium held at Hadassah Hospital

An all-day symposium on Advances in Brain Imaging with PET and fMRI was held at Hadassah Hospital, Ein Kerem, Jerusalem in February of 2016. The Symposium was organized as part of a Fulbright Fellowship for Teaching and Research to the Guest Editor (EDM). Funding and logistical support for the event were provided by Fulbright in Israel, Hadassah, the Radiology Department of Yale University, and a grant from the NIH for “International Research Collaboration on Drug Abuse and Addiction Research” (PA-12-041). Following the Hadassah Symposium, six of the invited speakers agreed to contribute review articles based on and expanding their lectures in Jerusalem. The six review articles make up this special issue.

## Imaging dopamine in addiction

In a thoughtful conceptual review of the neuroimaging of drug abuse, (speaker) David Kareken highlights the difference between reward from a drug and effort to obtain that drug. The author discusses preclinical studies that have been able to distinguish brain responses to the two phenomena and suggests that human imaging studies may be blurring the two responses together. Results from Kareken and his colleagues using PET to image the dopaminergic responses to alcohol or beer flavor are presented in support of the claim that effects of goal-directed motor behavior and reward-related cues are separate and (possibly) additive responses. The relevant pathways and their respective signals are nicely diagrammed. This paper serves to encourage future work and more refined designs of PET and fMRI neuroimaging experiments to advance our understanding of drugs of abuse and natural rewards.

Heather Liu and colleagues (speaker: Evan Morris) present the argument for the application of special kinetic models that allow for time-variation in neurotransmitter levels to describe dynamic PET data acquired in the presence of short-lived dopaminergic responses to drugs or behavioral challenges. In the course of their article, the authors review the literature of PET imaging of smoking cigarettes. The review seizes on an interesting ‘natural experiment’ that occurred in the PET literature: two nearly identical PET studies of the dopaminergic response to a stress task - analyzed in two different ways, to highlight the possible advantages of models that include time-varying terms or time-varying parameters. Time varying models in this context describe dynamic functions (‘movies’) of dopamine at each voxel rather than static parametric images. To support their claims, Liu et al., also present some findings from their own work using the ‘lpntPET’ model to uncover time-varying dopamine responses to smoking cigarettes that are present in the ventral striatum of male but not female smokers. Dopamine movies are available in the online article.

## From dopamine to serotonin: Parkinson’s and complications

Mikaeel Valli et al. (speaker: Antonio Strafella) review an extensive literature on neuroimaging of the non-motor complications of Parkinson’s disease (PD). PET, SPECT and fMRI studies have revealed anomalies of both function and structure in this patient population. The authors divide their review into studies of four major behavioral complications of PD: depression (dPD), apathy (aPD), impulse control disorders (ICD-PD) and visual hallucinations (VH-PD). As the authors explain, PET has been used to reveal both dopaminergic and serotonergic abnormalities in the various subtypes of PD compared to PD patients without complications. Both dopamine and serotonin are implicated in dPD and aPD, whereas thanks in large part to work by Strafella and colleagues, ICD-PD has been shown to be primarily dopaminergic (e.g., ICD-PD patients have increased dopamine release in response to reward cues and gambling tasks). In contrast, serotonergic dysfunction may be at the root cause of VH-PD. MRI has revealed instances of structural deficits – low gray matter in orbitofrontal cortex in ICD-PD, white matter degeneration in VH-PD. fMRI has been used to show diminished resting state functional connectivity (rsFC) to striatal regions in ICD-PD. The authors argue for the need for further investigations to flesh out what is already a complicated story and in particular for rsFC studies of PD with anxiety which are lacking.

## Neurodegeneration: Tau imaging

Cristina Lois et al. (speaker: Julie Price) review the current state of tau tracers for PET imaging of Alzheimers disease (AD). As the research community has come to appreciate the importance of tau deposition as an indicator of neuronal loss, neurodegeneration and cognitive decline in AD, the desire grows for a PET tracer to detect, monitor disease, and possibly monitor the action of anti-tau therapy. The authors review in detail the status of various tau tracers in development. The ideal tracer must cross the blood-brain barrier and cross the cell membrane (tau is an intracellular protein). It must have low off target binding, especially to amyloid-β, which co-locates with tau in some parts of the brain. It must have favorable kinetics of uptake so that a stable SUVR or V_T_ measurement can be made from the time-limited scan data. Whatever the calculated uptake measure, it must be reflective of cognitive decline. As of this writing, there is no FDA-approved tracer for tau but many have been examined (their limitations are detailed) and a new generation of tracers is under development and testing. Beyond general methodological and pharmacokinetic modeling considerations, the article is enhanced by informative PET images of AD brains at different stages of disease imaged with different tau tracers.

## Neurodegeneration: Amyloid imaging in Down syndrome

Patrick Lao and colleagues (speaker: Bradley Christian) discuss the special steps that must be taken to image amyloid-β in Down syndrome (trisomy 21). The gene for amyloid precursor protein (APP) resides on chromosome 21 which exists in triplicate in Down syndrome (DS). Thus, overproduction of amyloid plaques begins early in life and leads to a high prevalence of Alzheimer’s disease in individuals with DS (75% occurrence by age 65). Because these individuals also have known morphological anomalies (small cerebellum, frontal and temporal cortices, etc) and because they have difficulty remaining still in the MR scanner, the standard approach of (1) spatial normalization of an individual’s PET image to the same individual’s T1 MRI and then (2) normalization to a structural template made from T1 MRI images of healthy controls is suboptimal. Instead, the authors propose a two-step procedure (diagrammed with illustrative images) for creating a template of DS brains based directly on ^11^C-PIB images (of tracer binding to amyloid-β). The article details the procedure for creating the DS-specific template and the team’s initial attempts at validation.

## PET imaging in drug development: Occupancy to Alzheimer’s to Parkinson’s

Ivonne Suridjan and colleagues (speaker: Ilan Rabiner) provide an overview of the various roles played by PET imaging in CNS drug development. The authors, Pharma insiders, describe in detail how PET is used for biodistribution, occupancy, and for monitoring disease progression/regression. Biodistribution is the regional uptake of a radiolabelled version of the drug. At a minimum, it can confirm that a drug does or does not enter the target organ (i.e., the brain for CNS drugs) in vivo. Rules of thumb for high and low-penetrant drugs are provided in one of many useful tables. The authors speculate that PET imaging of novel biologics (e.g., antibodies) may require labeling with longer-lived isotopes to accommodate longer biological half-lives and novel models to describe brain uptake that occurs by means other than passive diffusion. Receptor-occupancy (RO) studies are intended to quantify target engagement of the unlabelled drug. These studies require a selective PET tracer to be blocked or displaced from the target. An extensive table of tracers and their targets is also provided. RO studies can also help to determine the desired dose range if the desired occupancy of the target to achieve a therapeutic effect is already known. Again, rules of thumb are provided in tabular form for interpreting RO results. Finally, the article returns to topics covered by others. Imaging amyloid and tau for Alzheimer’s and dopamine receptors and transporters for Parkinson’s. These examples serve to highlight the strengths and weaknesses of current PET tracers for monitoring disease progression, for selecting patients for clinical trials, and for monitoring the efficacy of drugs to stem or reverse the pathological deposition of proteins.

